# Glycoprotein nonmetastatic melanoma protein B extracellular fragment shows neuroprotective effects and activates the PI3K/Akt and MEK/ERK pathways via the Na^+^/K^+^-ATPase

**DOI:** 10.1038/srep23241

**Published:** 2016-03-18

**Authors:** Yoko Ono, Kazuhiro Tsuruma, Masafumi Takata, Masamitsu Shimazawa, Hideaki Hara

**Affiliations:** 1 Molecular Pharmacology, Department of Biofunctional Evaluation, Gifu Pharmaceutical University, Gifu, Japan

## Abstract

Glycoprotein nonmetastatic melanoma protein B (GPNMB) plays important roles in various types of cancer and amyotrophic lateral sclerosis (ALS). The details of GPNMB function and its interacting protein have not been clarified. Therefore, to identify GPNMB binding partners on the cell membrane, we used membrane protein library/BLOTCHIP-MS technology, which enables us to analyze all cell membrane proteins as binding partners of the GPNMB extracellular fragment. As a result of a comprehensive search, we identified the alpha subunits of Na^+^/K^+^-ATPase (NKA) as a possible binding partner. We confirmed the interaction between the GPNMB extracellular fragment and NKA by immunoprecipitation and immunostaining in NSC-34 cells. Indeed, endogenous GPNMB extracellular fragment bound to and colocalized with NKA alpha subunits. Furthermore, exogenous GPNMB extracellular fragment, i.e., human recombinant GPNMB, also bound to and colocalized with NKA alpha subunits. Additionally, we found that the GPNMB extracellular fragment had neuroprotective effects and activated the phosphoinositide 3-kinase (PI3K)/Akt and mitogen-activated protein kinase (MAPK)-extracellular signal-regulated kinase (ERK) kinase (MEK)/ERK pathways via NKA. These findings indicated that NKA may act as a novel “receptor” for the GPNMB extracellular fragment, offering additional molecular targets for the treatment of GPNMB-related diseases, including various types of cancer and ALS.

Glycoprotein nonmetastatic melanoma protein B (GPNMB) is a type I transmembrane protein and is also known as osteoactivin, dendritic cell-heparin integrin ligand (DC-HIL), and hematopoietic growth factor inducible neurokinin-1 type^1^,^2^,^3^. GPNMB is composed of three domains, including a long extracellular domain (ECD), a single transmembrane domain, and a relatively short cytoplasmic tail^4^. The ECD has an arginine-glycine-aspartic acid (RGD) motif that is required for the GPNMB adhesive^5^,^6^ and a polycystic kidney disease (PKD) domain whose function in GPNMB is unidentified^7^.

GPNMB was initially cloned from poorly metastatic melanoma cells as a regulator of tumor growth^8^. GPNMB is important for the invasion and metastasis of several cancers, such as breast cancer^9^, colorectal cancer^10^, hepatocellular carcinoma, and cutaneous melanoma^11^. In addition, GPNMB plays diverse roles in normal cells, such as T-cell activation and promoting the specialization of osteoclasts and osteoblasts^12^,^13^. Moreover, we have discovered that high GPNMB protein levels were observed in the cerebrospinal fluid (CSF), sera, and spinal cords of human patients with amyotrophic lateral sclerosis (ALS)^14^.

A number of types I transmembrane proteins are activated after cleavage into extracellular and intracellular fragments by a disintegrin and metalloproteases (ADAMs) or γ-secretase^15^. GPNMB is also cleaved by ADAM10, resulting in activation^16^. The extracellular fragment of GPNMB enhances ERK phosphorylation along with upregulating matrix metalloproteinase-3 (MMP-3) expression and induces endothelial cell migration^17^. We reported that GPNMB was cleaved and secreted extracellularly, and the extracellular fragment of GPNMB had protective effects against mutant superoxide dismutase 1 (SOD1)-induced neurotoxicity via the activation of the phosphoinositide-3-kinase (PI3K)/Akt and MEK/ERK pathways^14^. Therefore, we hypothesized that the extracellular fragment of GPNMB binds a receptor or proteins on the plasma membrane, and then activates the PI3K/Akt and MEK/ERK pathways. Here, we have identified the Na^+^/K^+^-ATPase as a receptor for the extracellular fragment of GPNMB that mediates activation of cellular signaling pathways and subsequent neuroprotective effects.

## Results

### Identification of binding partners for the GPNMB extracellular fragment

**Figure 1:**
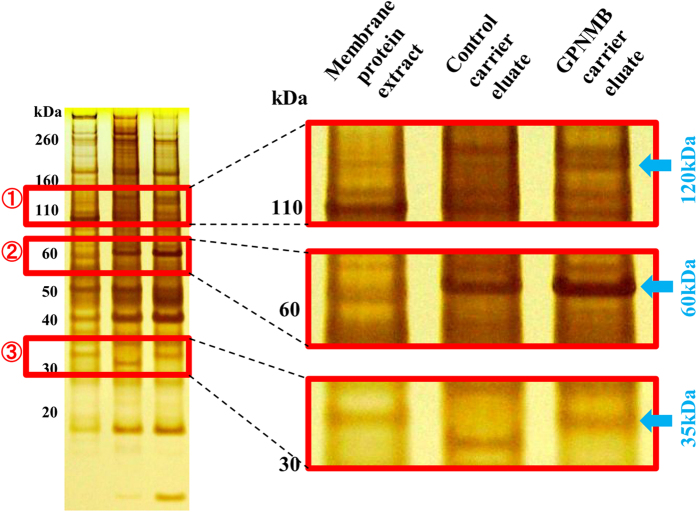
Identification of binding partners for the GPNMB extracellular fragment. To identify novel interacting proteins of GPNMB, we conducted a comprehensive search using Membrane Protein Library (MPL)/BLOTCHIP-MS technology. MPL enables users to extract all cellular membrane proteins and reconstitute them in artificial lipid bilayers composed of phospholipid liposomes to protein function. By differential analysis of SDS-PAGE electrophoresis profile the MPLs, three bands were detected at about 35, 60, and 120 kDa and were considered possible receptors.

To identify novel cellular membrane receptors or proteins that interact with the extracellular fragment of GPNMB, we conducted a comprehensive search using Membrane Protein Library (MPL)/BLOTCHIP-MS technology (Protosera Inc.)^18^,^19^. Traditional technologies can treat only one membrane protein at a time and do not allow for systematic or comprehensive screening. On the other hand, the MPL enabled us to extract all the membrane proteins by reconstituting the proteins in artificial lipid bilayers made of phospholipid liposomes that preserve protein function. Therefore, the MPL/BLOTCHIP-MS technology enabled us to analyze all cell membrane proteins as binding partner of the GPNMB extracellular fragment. The MPL was prepared by fusing the membrane fraction of a cellular lysate with liposomes. Then, the MPL was bound to the GPNMB extracellular fragment immobilization carrier or control carrier, and the MPL binding carrier was eluted. Sodium dodecyl sulfate-polyacrylamide gel electrophoresis (SDS-PAGE) of the MPL complex with the extracellular fragment of GPNMB revealed the presence of three bands at about 35, 60, and 120 kDa (Fig. 1) compared with the control. Next, we identified novel binding proteins of GPNMB by analysis of the bands using matrix assisted laser desorption/ionization-time of flight-mass spectrometry (MALDI-TOF-MS) or liquid chromatography-transform- mass spectrometry (LC-FT-MS). The results indicated that the alpha subunits of Na^+^/K^+^-ATPase (NKA) or the voltage-dependent anion channel 1 (VDAC1) were GPNMB binding partners (Table 1). NKA is localized to the plasma membrane in all mammalian cells^20^, whereas VDAC1 is localized primarily to the mitochondria membrane^21^. When starting this study, we hypothesized that the extracellular fragment of GPNMB secreted may bind to a plasma membrane protein acting as a receptor. Therefore, we focused our efforts on determining whether NKA was a receptor for the extracellular fragment of GPNMB because NKA exists on plasma membrane, whereas VDAC1 does not.

### The extracellular fragment of GPNMB interacted and colocalized with the NKA α1 and α3 subunits in NSC-34 cells murine motor neuron

**Figure 2:**
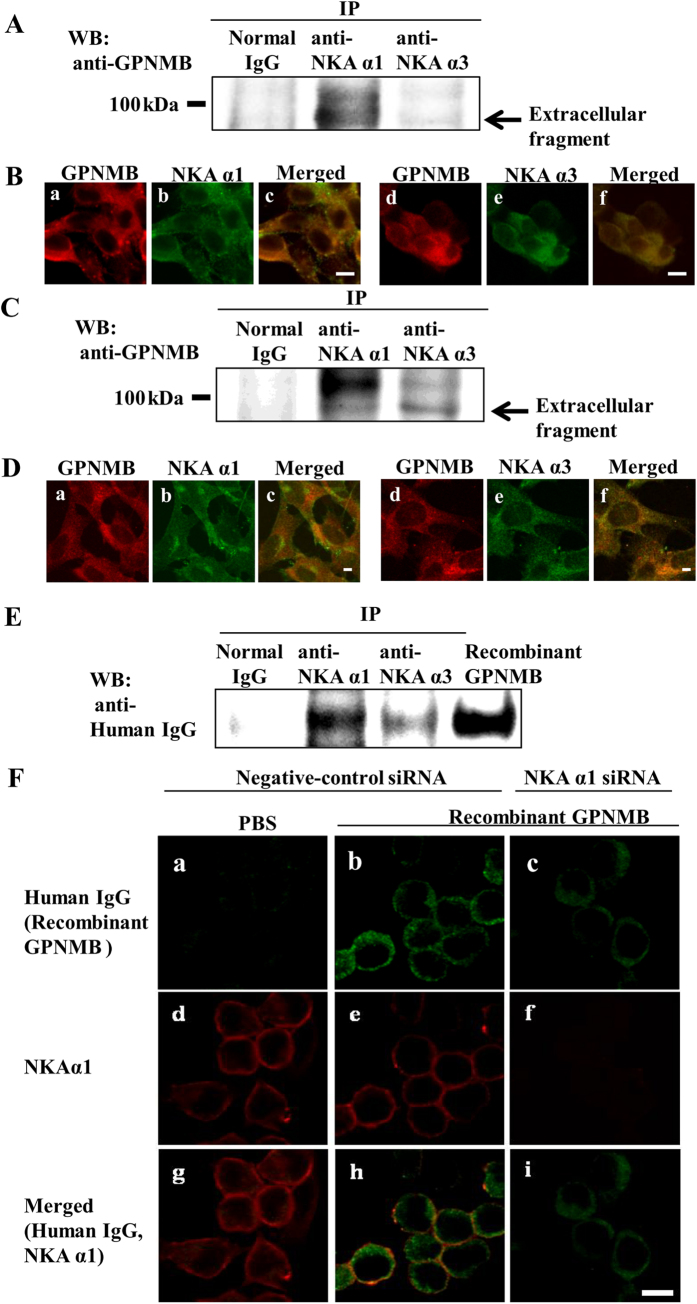
The extracellular fragment of GPNMB interacted and colocalized with the NKA α1 and α3 subunits in NSC-34 murine motor neuron cells. (**A**) An equal amount of cell lysate was subjected immunoprecipitation (IP) by anti-NKA α1 antibody, anti-NKA α3 antibody, or normal mouse IgG (Normal IgG), and detected by western blot using anti-GPNMB antibody. (**B**) Co-immunostaining was conducted to localize NAK and GPNMB by anti-GPNMB antibody (red: a and d) and anti-NAK α1 antibody (green: b), or anti-NAK α3 antibody (green: e). (**C**) 661 W cells were plated at 2 × 10^5 ^cells/well in 6-well plates and incubated overnight. The cell lysate was subjected immunoprecipitation (IP) by anti-NKA α1 antibody, anti-NKA α3 antibody, or normal mouse IgG (Normal IgG), and detected by western blot using anti-GPNMB antibody. (**D**) 661 W cells were seeded at 5 × 10^4 ^cells/well in a Lab-Tek II Chamber slide (Thermo Fisher Scientific) and incubated overnight. Co-immunostaining in 661 W cell was conducted to localize NAK and GPNMB by anti-GPNMB antibody (red: a and d) and anti-NAK α1antibody (green: b), or anti-NAK α3 antibody (green: e). (**E**) NSC-34 cells were pretreated with human recombinant GPNMB and subjected to IP by anti-NAK α1 antibody, anti-NAK α3 antibody, or normal mouse IgG (Normal IgG), and detected by western blot with anti-human IgG antibody. (**F**) Co-immunostaining was conducted to localize of human recombinant GPNMB (green: a, b, and c) and NAK α1 (red: d, e, and f) in NSC-34 cells. The cells were pretreated with negative-control siRNA, and then treated with PBS (a, d, and g) or human recombinant GPNMB (b, e, and h). The cells were pretreated with NAK α1 siRNA, and then treated with the human recombinant GPNMB (c, f, and i). IP: immunoprecipitation, WB: Western blotting. Scale bar = 5 μm.

To determine whether the endogenous extracellular fragment of GPNMB interacted with NKA under cellular conditions, we performed immunoprecipitation and co-immunostaining in NSC-34 cells under normal conditions. Cell lysates were immunoprecipitated by anti-NKA α1 or anti-NKA α3 antibodies and detected using anti-GPNMB antibodies. Western blot results indicated that the endogenous extracellular fragment of GPNMB bound to NKA α1 and NKA α3 (Fig. 2A). Next, we performed co-immunostaining using anti-GPNMB antibodies and anti-NKA α1 or anti-NKA α3 antibodies. Our results showed that GPNMB colocalized with both of the NKA alpha subunits (Fig. 2B). Additionally, to confirm whether the extracellular fragment of GPNMB interacts with NKA alpha subunit in another cell line, the photoreceptor-derived cell line 661 W, we performed the immunopresipitation and co-immunostaininng in the 661 W cells under normal condition. 661 W cell lysates were also immunoprecipitated by anti-NKA α1 or anti-NKA α3 antibodies and detected using anti-GPNMB antibodies. Western blot results indicated that the endogenous extracellular fragment of GPNMB bound to NKA α1 and NKA α3 (Fig. 2C). Moreover, co-immunostaining using anti-GPNMB antibodies and anti-NKA α1 or anti-NKA α3 antibodies showed that GPNMB colocalized with both of the NKA α1 and α3 subunits in 661 W cells. These results indicate that the endogenous extracellular fragment of GPNMB interacts with NKA α1 and α3 subunits in 661 W cells.

Next, we investigated whether the exogenous GPNMB extracellular fragment interacted with the NKA alpha subunits by immunoprecipitation and co-immunostaining. NSC-34 cells were exogenously treated with the human recombinant GPNMB extracellular fragment (2.5 μg/ml), followed by immunoprecipitation with anti-NKA α1 or anti-NKA α3 antibodies and detection of human recombinant GPNMB (recombinant human GPNMB Fc chimera protein), which contains human IgG, using anti-human IgG anibodies. If the anti-GPNMB antibody was used to detect human recombinant GPNMB, it may not be able to distinguish recombinant GPNMB from endogenous GPNMB because the antibody may recognize both endogenous and exogenous GPNMB. Therefore, we used anti-human IgG antibodies to detect only human recombinant GPNMB. Additionally, we chose 2.5 μg/ml human recombinant GPNMB for immunoprecipitation and immunostaining because this concentration has been used to investigate the neuroprotective effects of the protein and the activation of survival signaling pathways in our previous report^14^. Western blot results indicated that the human recombinant GPNMB was bound to NKA α1 and NKA α3 in NSC-34 cells (Fig. 2E).

To determine the specificity of the interaction of GPNMB with NKA, cells were pretreated with NKA α1 small-interfering RNA (siRNA) or negative-control siRNA. The human recombinant GPNMB partially colocalized with NKA α1 on the plasma membrane of cells transfected with negative-control siRNA (Fig. 2F). Interestingly, the human recombinant GPNMB localized not only on the cell membrane, but also in the cytoplasm. On the other hand, the fluorescence was attenuated by NKA α1 siRNA transfection. Together, these results suggested that the endogenous and exogenous GPNMB extracellular fragments interact with NKA alpha subunits.

### The extracellular fragment of GPNMB changed the cellular membrane potential via NKA

**Figure 3:**
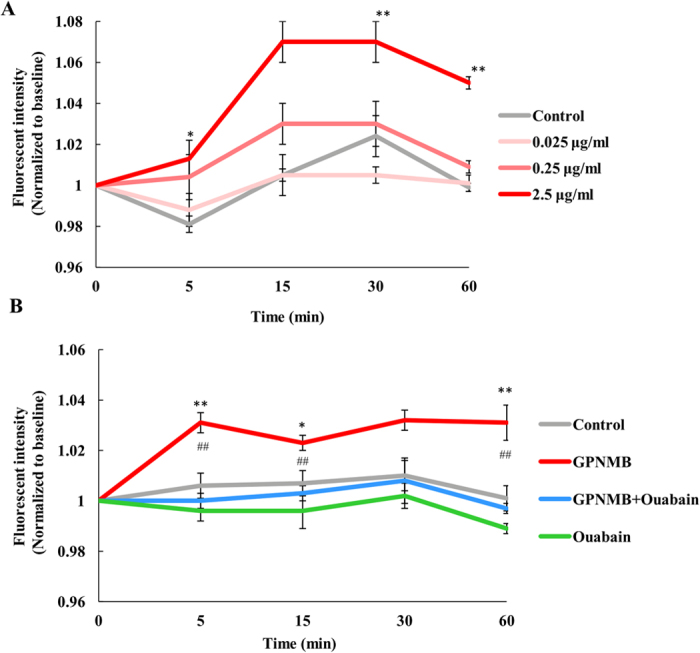
The extracellular fragment of GPNMB changed the cellular membrane potential via NKA. NSC-34 cells were loaded with the membrane potential dye, HLB021-152. The fluorescence signal of HLB021-152 was measured using microplate reader before 5, 15, 30, and 60 min after adding human recombinant GPNMB (F_0_) or ouabain (F). The ratio F/F_0_ was calculated. (**A**) NSC-34 cells were treated with the human recombinant GPNMB (0.025, 0.25, and 2.5 μg/ml). ^*^P < 0.05, ^**^P < 0.01 (control group *vs*. GPNMB group, Dunnett’s test). Each column and bar represents the mean ± S.E.M. (n = 5). (**B**) NSC-34 cells were treated with human recombinant GPNMB (2.5 μg/ml) or ouabain, an NAK inhibitor (10 μM). *P < 0.05, **P < 0.01 (control group *vs*. GPNMB group), ^##^*P* < 0.01 (GPNMB group *vs*. ouabain + GPNMB group, Tukey’s test). Each column and bar represents the mean ± S.E.M. (n = 5).

NKA is the major transporter of Na^+^ and K^+^ across the plasma membranes of most mammalian cells and maintains high K^+^ and low Na^+^ concentrations in the cytoplasm^22^. If the extracellular fragment of GPNMB stimulates signaling via the NKA, the cellular membrane potential may be changed in response to GPNMB binding to NKA. Therefore, we investigated the membrane potential change using HLB021-152, a fluorescent probe for cellular membrane potential. When the cellular membrane potential is changed, HLB021-152 is taken up by the cell and binds to intracellular membranes and proteins, which causes the fluorescence intensity to increase^23^. First, we investigated the appropriate concentration of recombinant GPNMB (0.025–2.5 μg/ml) for altering change the cellular membrane potential. The results showed that 2.5 μg/ml human recombinant GPNMB increased the intensity of fluorescence, that is to say, changed the cell membrane potential (Fig. 3A). This concentration of recombinant GPNMB corresponded with the concentration that significantly protected NSC-34 motor neuron cells against SOD1^G93A^ -induced cell death and activated the PI3K/Akt and MEK/ERK pathways in our previous report^14^.

Next, to confirm that the effects of GPNMB on the cellular membrane potential were mediated by NKA, we investigated whether the change in cellular membrane potential was blocked by the NKA inhibitor ouabain. The intensity of fluorescence increased initially after stimulation with 2.5 μg/ml human recombinant GPNMB; however, this increase was blocked by ouabain (Fig. 3B). This suggests that the extracellular fragment of GPNMB may activate NKA by binding to NKA.

### The neuroprotective effect of the GPNMB extracellular fragment against SOD1^G93A^ with serum free stress-induced cell death was blocked by NKA inhibitors

**Figure 4:**
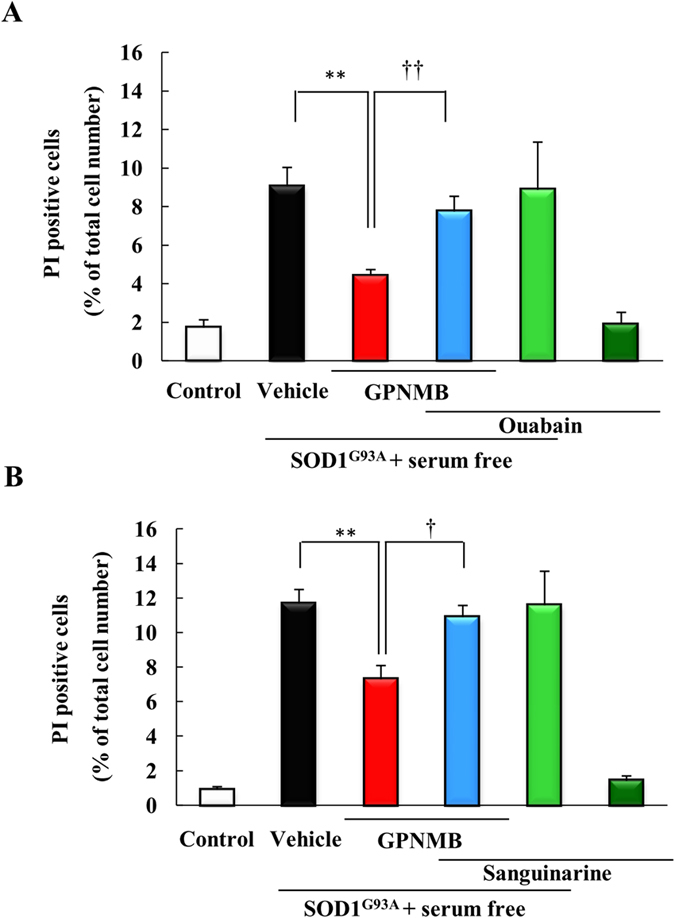
The neuroprotective effect of the GPNMB extracellular fragment against SOD1^G93A^ with serum free stress-induced cell death was blocked by NKA inhibitors. Cells were treated with vehicle or with human recombinant GPNMB (2.5 μg/ml), along with NAK inhibitors, (**A**) ouabain (10 μM) and (**B**) sanguinarine (0.3 μM), concomitantly with removing the serum. The number of cells displaying PI or Hoechst 33342 fluorescence was counted, and the cell death rate was expressed as the ratio of PI-positive cells to Hoechst 33342-positive cells. ^##^*p* < 0.01 *vs*. control group (Student’s *t*-test). **p < 0.01 *vs*. Vehicle group, ^††^*p* < 0.01 *vs*. GPNMB group (Student’s *t*-test). Each column and bar represents the mean ± S.E.M. (n = 4). Scale bar = 50 μm.

Previously, we have reported that the extracellular fragment of GPNMB protected NSC-34 murine motor neuron cells against SOD1^G93A^ and serum free stress-induced cell death, which served as an *in vitro* model of ALS^14^. If the NKA is a receptor of GPNMB, we hypothesized that NKA inhibitors might diminish the neuroprotective effect of GPNMB. We investigated the effect of two NKA inhibitors, ouabain and sanguinarine, on the neuroprotective effect of GPNMB. When cells were co-treated ouabain (10 μM) or sanguinarine (0.3 μM) and human recombinant GPNMB (2.5 μg/ml), the NKA inhibitors were not cytotoxic at the concentrations used. The two NKA inhibitors obstructed the neuroprotective effect of human recombinant GPNMB against SOD1^G93A^ and serum free stress-induced cell death (Fig. 4A,B). These results indicate that the extracellular fragment of GPNMB may exert its neuroprotective effect via NKA binding.

### Activation of the PI3K/Akt and MEK/ERK pathways by the extracellular fragment of GPNMB was blocked by NKA inhibitors

**Figure 5:**
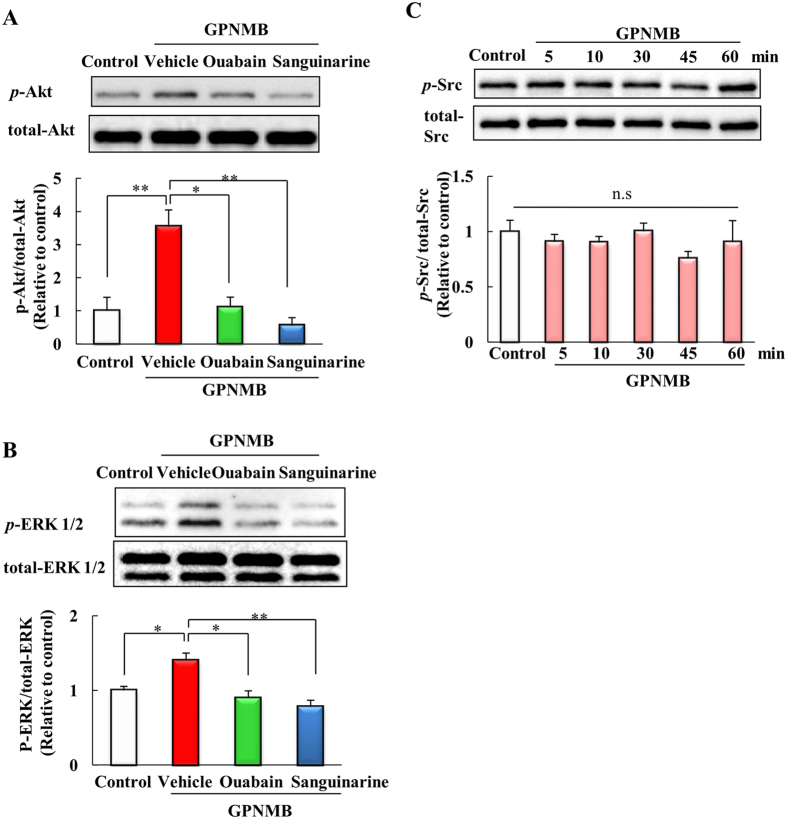
Activation of the PI3K/Akt and MEK/ERK pathways by the extracellular fragment of GPNMB was blocked by NKA inhibitors. NSC34 cells were treated with human recombinant GPNMB (2.5 μg/ml), and ouabain (10 μM) or sanguinarine (0.3 μM). (**A**) The level of phosphorylated Akt was quantified relative to total Akt. (**B**) The level of phosphorylated ERK1/2 was quantified relative to total ERK. Each column represents the mean ± S.E.M. (n = 3 or 4). *p < 0.05, **p < 0.01 *vs*. vehicle group (Tukey’s test). (**C**) The level of *p*-Src protein was examined at different time points (0–60 min) after incubating with the recombinant extracellular GPNMB (2.5 μg/ml). The level of phosphorylated Src was quantified relative to total Src. Statistical comparisons were made using Dunnett’s test (*vs*. control group). Each column and bar represents the mean ± S.E.M. (n = 4). n.s.: not significant.

The extracellular fragment of GPNMB activates the PI3K/Akt and MEK/ERK pathways^14^,^17^. We investigated whether NKA inhibitors might block the activation of these pathways. Cells were co-treated ouabain (10 μM) or sanguinarine (0.3 μM), and human recombinant GPNMB (2.5 μg/ml). The human recombinant GPNMB induced increases in phosphorylated-Akt and phosphorylated-ERK levels, consistent with previous reports, and NKA inhibitors blocked the increase in phosphorylated-Akt and phosphorylated-ERK (Fig. 5A,B). These results indicate that the extracellular fragment of GPNMB may activate the PI3K/Akt and MEK/ERK pathways via the NKA. We also investigated the activation of Src, which is downstream of the NKA. However, the GPNMB extracellular fragment did not affect Src phosphorylation (Fig. 5C).

### The neuroprotective effect of GPNMB extracellular fragment against SOD1^G93A^ with serum free-induced cell death was inhibited by NKA α1 siRNA

**Figure 6:**
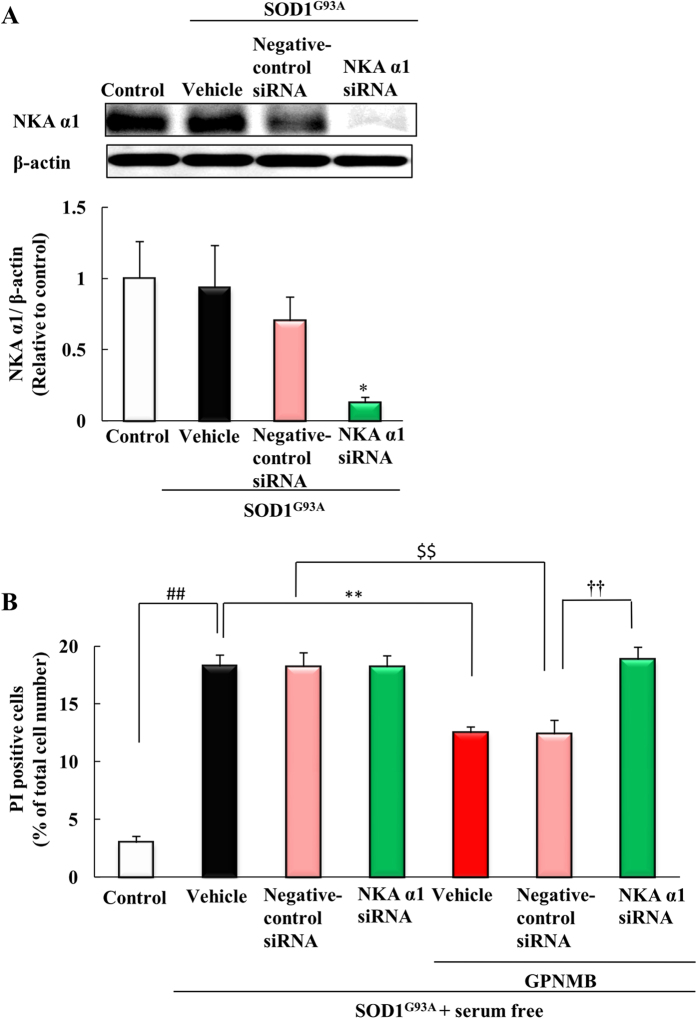
The neuroprotective effect of GPNMB extracellular fragment against SOD1^G93A^ with serum free-induced cell death was inhibited by NKA α1 siRNA. (**A**) Cells were transfected with the empty vector (mock) as a control or with human mutant SOD1^G93A^ cDNAs and mouse NKA α1 siRNA or negative-control siRNA. Western blotting was used to confirm that NKA α1 levels were knocked down. *p < 0.05 vs. negative-control group (Student’s t-test). Each column and bar represents the mean ± S.E.M. (n = 6). (**B**) Cells were treated with vehicle or with human recombinant GPNMB (2.5 μg/ml) concomitantly with removing the serum. The number of cells displaying PI or Hoechst 33342 fluorescence was counted, and the cell death rate was expressed as the ratio of PI-positive cells to Hoechst 33342-positive cells. ^##^*p* < 0.01 vs. control group (Student’s t-test). **p < 0.01 vs. vehicle group, ^††^*p* < 0.01 vs GPNMB treated group (Tukey’s test). Each column and bar represents the mean ± S.E.M. (n = 4).

To clarify the involvement of NKA in the neuroprotective effect of GPNMB extracellular fragment, we used NKA α1 siRNA to knockdown NKA α1 protein levels. NSC-34 cells were transfected with the empty vector (mock) or human mutant SOD1^G93A^ cDNAs and mouse NKA α1 siRNA or negative-control siRNA. The knockdown level of the NKA α1 was confirmed by western blot analysis (Fig. 6A), and NKA α1 siRNA significantly reduced the amount of NKA α1 protein. Next, we conducted a cell death assay against SOD1^G93A^ with serum free stress-induced cell death. Transfection of NKA α1 siRNA or negative-control siRNA did not affect the rate of cell death induced by SOD1^G93A^ with serum free stress. The human recombinant GPNMB (2.5 μg/ml) protected cells against SOD1^G93A^ with serum free stress-induced cell death when cells were transfected with negative-control siRNA. In contrast, in the group transfected with NKA α1 siRNA, the human recombinant GPNMB did not produce protective effects (Fig. 6B), suggesting that at least the NKA α1 participates in the neuroprotective effect of human recombinant GPNMB.

### Activation of the PI3K/Akt and MEK/ERK pathways by the extracellular fragment of GPNMB was blocked by NKA α1 siRNA

**Figure 7:**
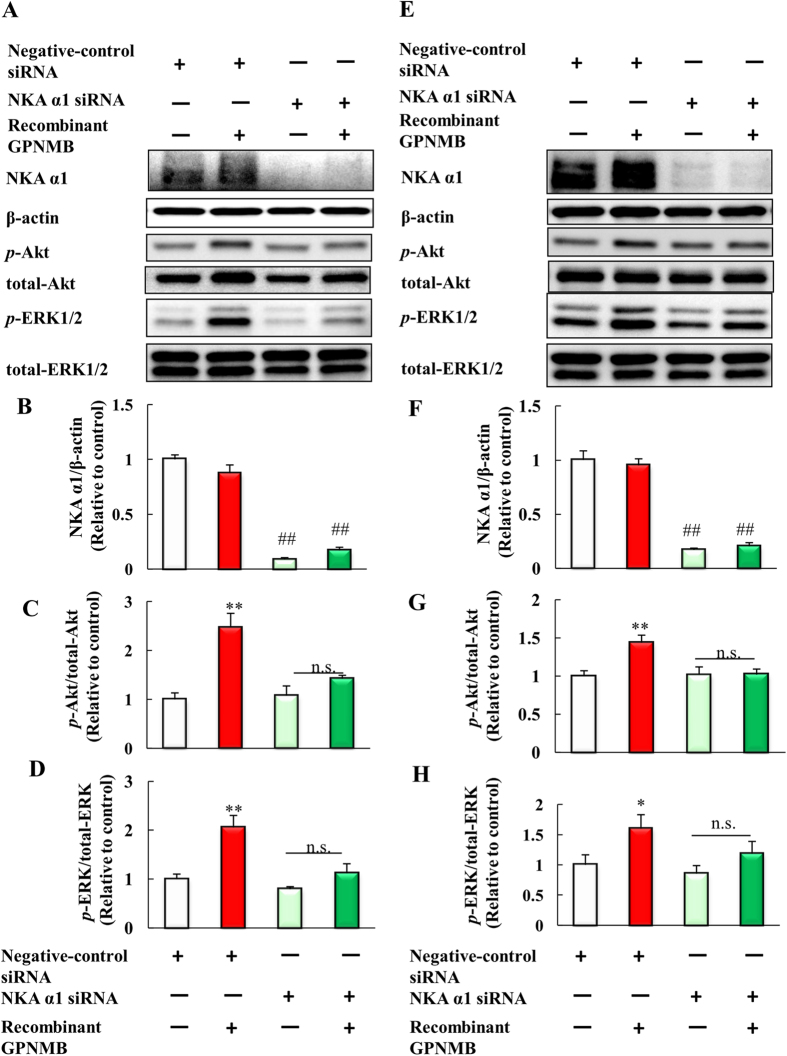
Activation of the PI3K/Akt and MEK/ERK pathways by the extracellular fragment of GPNMB was blocked by NKA α1 siRNA. (**A**) NSC34 cells were transfected with mouse NKA α1 siRNA or negative-control siRNA, and then treated with the human recombinant GPNMB (2.5 μg/ml). Western blotting analyses of NKA α1, β-actin, phosphorylated-Akt, total-Akt, phosphorylated-ERK1/2, and total-ERK1/2. (**B**) The protein levels of NKA α1 were quantified relative to β-actin. (**C**) The protein levels of phosphorylated-Akt were quantified relative to total-Akt. (**D**) The protein levels of phosphorylated-ERK1/2 were quantified relative to total-ERK1/2. Each column represents the mean ± S.E.M. (n = 4). n.s.: not significant. ^##^*p* < 0.01 *vs*. negative-control, **p < 0.01 *vs*. GPNMB treated group (Student’s *t*-test). (**E**) 661W cells were plated at 1.5 × 10^4 ^cells/well in 24-well plates and incubated overnight. Negative-control siRNA and NKA α1 siRNA transfected for 24 h, and then cells were treated with PBS or human recombinant GPNMB. Western blotting analyses of NKA α1, β-actin, phosphorylated-Akt, total-Akt, phosphorylated-ERK1/2, and total-ERK1/2. (**F**) The protein levels of NKA α1 were quantified relative to β-actin. (**G**) The protein levels of phosphorylated-Akt were quantified relative to total-Akt. (**H**) The protein levels of phosphorylated-ERK1/2 were quantified relative to total-ERK1/2. Each column represents the mean ± S.E.M. (n = 4 or 5). n.s.: not significant. ^##^*p* < 0.01 *vs*. negative-control, *p < 0.05, **p < 0.01 *vs*. GPNMB treated group (Student’s *t*-test).

Next, we investigated whether knockdown of NKA α1 affected the activation of PI3K/Akt and MEK/ERK pathways by the extracellular fragment of GPNMB. NSC-34 cells were transfected with NKA α1 siRNA or negative-control siRNA, and then these cells were treated with human recombinant GPNMB (2.5 μg/ml). NKA α1 proteins were adequately knocked down by NKA α1 siRNA (Fig. 7A,B). In the cells transfected with negative-control siRNA, the human recombinant GPNMB upregulated the levels of phosphorylated-Akt and phosphorylated-ERK. On the other hand, NKA α1 knockdown attenuated the effect of GPNMB extracellular fragment on the phosphorylation of Akt and ERK (Fig. 7A,C,D). Moreover, we also investigated whether extracellular fragment of GPNMB activates PI3K/Akt and MEK/ERK pathways via NKA α1 in 661 W cell line. In the 661 W cells, NKA α1 proteins were adequately knocked down by NKA α1 siRNA (Fig. 7E,F). In the 661 W cells as well as NSC-34 cells, the human recombinant GPNMB upregulated the levels of phosphorylated-Akt and phosphorylated-ERK, and NKA α1 knockdown attenuated the effect of GPNMB extracellular fragment on the phosphorylation of Akt and ERK (Fig 7E,G,H). Together, these results suggest that the extracellular fragment of GPNMB activates the PI3K/Akt and MEK/ERK pathways through the NKA α1.

## Discussion

In the present study, we identified the alpha subunits of NKA as binding partners of the GPNMB extracellular fragment by a comprehensive search of receptors or protein on the cell membrane using MPL/BLOTCHIP-MS technology, as described previously^18^,^19^. NKA is the major transporter of Na^+^ and K^+^ across the plasma membrane of most mammalian cells and maintains the high K^+^ and low Na^+^ concentrations in the cytoplasm^20^,^22^. The NKA α1 and NKA α3 subunits are significantly increased in melanoma^24^, glioblastoma^25^, renal cell carcinoma^26^, lung cancers^27^, colorectal cancers^28^, and hepatocellular carcinomas^29^. Moreover, the global cellular distribution of NKA is decreased in an SOD1^G93A^ transgenic mouse model of ALS^30^. These reports indicate that function of NKA is important in the pathology of cancer and/or ALS. GPNMB is also important for the invasion and metastasis of several cancers, and has protective effects against ALS^4^,^14^. Moreover, GPNMB appears to work in close association with NKA. Therefore, we focused our efforts on determining whether NKA worked as a receptor for the extracellular fragment of GPNMB. Because NKA primarily functions as an ion pump, it was not clear whether NKA could also play a role as a receptor. However, multiple reports have shown that NKA has functions other than ion transport, and various ligands bind to and regulate NKA. For example, klotho, a transmembrane protein with particularly strong expression in the kidneys, binds to NKA and induces NKA activity^31^. Retinoschisin, the protein involved in the pathogenesis of X-linked juvenile retinoschisis, also binds to NKA and induces the activation of sterile α and TIR motif-containing 1 protein (SARM1)^32^. NKA has been suggested to function as a noncanonical cardiotonic steroid-binding receptor and activates the PI3K/Akt and MEK/ERK pathways^33^,^34^.

In our immunoprecipitation and immunostaining experiments, the concentration of human recombinant GPNMB extracellular fragment was 2.5 μg/ml; this concentration altered the cell membrane potential, had neuroprotective effects, and activated the PI3K/Akt and MEK/ERK pathways. In our previous report, the amounts of GPNMB secreted into the cerebrospinal fluid (CSF) were 1.3 ± 0.47 ng/ml in control patients and 2.3 ± 0.95 ng/ml in patients with sporadic ALS. Additionally, the amount of GPNMB secreted into the serum were 27.6 ± 7.1 ng/ml in control patients and 40.2 ± 9.1 ng/ml in patients with sporadic ALS^14^. The concentration used in this study was higher than the amount of GPNMB in the CSF or serum. However, GPNMB is highly expressed in motor neurons and astrocytes in the spinal cord. Therefore, 2.5 μg/ml recombinant GPNMB may be sufficient for binding to alpha subunits of NKA.

Both NKA inhibitors and NKA α1 siRNA blocked the neuroprotective effects of GPNMB against stress-induced cell death and activation of the PI3K/Akt and MEK/ERK pathways via GPNMB binding. Unexpectedly, NKA α1 siRNA transfection caused slight increases in the phosphorylation of Akt and ERK in the recombinant GPNMB-treated group, but not to the extent that occurred when cells were transfected with negative-control siRNA. These results suggested that the PI3K/Akt and MEK/ERK pathways may be slightly activated by recombinant GPNMB via the remaining NKAα1 after siRNA transfection because the siRNA was not 100% efficient. Additionally, the NKA α3 subunit may also be involved in the PI3K/Akt and MEK/ERK pathways, because NKA α3 was not knocked down in this examination. In any case, these results suggested that the extracellular fragment of GPNMB had neuroprotective effects and activated the PI3K/Akt and MEK/ERK pathways via NKA, supporting the possibility that NKA may act a novel receptor of the GPNMB extracellular fragment.

We also investigated the downstream signaling components involved in propagating a signal from GPNMB through NKA to the PI3K/Akt pathway or MEK/ERK pathway. Many reports have suggested that Src facilitates communication between NKA and the PI3K/Akt or MEK/ERK pathways. Src activation through NKA can occur through cardiotonic steroids (CTSs) and vertebrate-derived aglycones^35^, followed by activation of sperm epidermal growth factor receptor (EGFR) and various downstream effectors, including the PI3K/Akt or MEK/ERK pathways^36^,^37^,^38^. Therefore, we investigated whether the extracellular fragment of GPNMB activated Src. However, our results showed that GPNMB did not increase Src phosphorylation, suggesting that Src was not involved in signaling by GPNMB. GPNMB enhances bone regeneration by regulating fibroblast growth factor receptor (FGFR) signaling^39^. FGFR also activates various downstream effectors, including the MAPK and PI3K-Akt-PDPK2 pathways^40^,^41^,^42^. In addition, NKA participates in FGF-2 release^43^,^44^, and FGF-2 binds to FGFR and promotes the MEK/ERK phosphorylation via FGFR signaling^45^. Together, these reports suggest that the activation of PI3K/Akt and MEK/ERK pathways by the extracellular fragment of GPNMB may be associated with FGFR signaling via NKA.

The extracellular fragment of GPNMB has an RGD motif, which is a cell adhesion motif displayed on many extracellular matrix and plasma proteins, and is involved in binding to various proteins through the RGD motif 5,6. Therefore, the RGD motif of the extracellular fragment of GPNMB may bind to the extracellular domain of NKA α1, which would then lead to further signaling through the PI3K/Akt and MEK/ERK pathways, resulting in neuroprotective effects. Further studies will be required to identify GPNMB extracellular fragment-specific binding sites and to investigate the interaction between GPNMB and NKA *in vivo*.

In conclusion, we have shown that the extracellular fragment of GPNMB associates directly with NKA alpha subunits. Therefore, we suggest that the NKA may function as a novel “receptor” of the GPNMB extracellular fragment and as an additional therapeutic target for various cancers and/or ALS.

## Materials and Methods

### Ligand and receptor discovery

To identify novel binding proteins of GPNMB, we used MPL/BLOTCHIP-MS technology (Protosera Inc., Hyogo, Japan)^18^,^19^. This method allowed us to analyze all the membrane proteins in the cell and organelle membranes. MPL was prepared by fusing the membrane fraction of a cellular lysate from mice whole brains of mice with liposomes. The extracellular fragment of GPNMB was immobilized with N-hydroxysuccinimide-activated sepharose 4 fast flow (S4F). After the immobilization reaction, all the contents were placed in an empty column, and the solution was stored as a flow-through fraction. The solution was blocked with 0.1 M Tris-Cl (pH 8.5), and the GPNMB extracellular fragment immobilization carrier (GPNMB-S4F) was obtained. A carrier subjected only to blocking was prepared as a control immobilization carrier (C-S4F). GPNMB-S4F or C-S4F was added into the MPL, and then the MPL was bound to ligand. Unbound MPLs were washed away and the targeted receptors or protein dominant MPLs were eluted. The elution was separated by electrophoresis. Protein bands were detected through the differential analysis of SDS-PAGE profiles of the MPLs and were identified using MALDI-TOF-MS or LC-FT-MS.

### Cell culture and transfection

NSC-34 cells, a neuroblastoma and spinal cord hybrid cell line, were purchased from Cosmo Bio Co., Ltd. (Tokyo, Japan). NSC-34 cells were maintained in Dulbecco’s modified Eagle’s medium (DMEM) with high glucose (Sigma-Aldrich, St. Louis, MO, USA) containing 10% fetal bovine serum (FBS; Valeant, Costa Mesa, CA, USA), 100 U/ml penicillin and 100 μg/ml streptomycin (Meiji Co. Ltd., Tokyo, Japan) in a humidified atmosphere containing 5% CO_2_ at 37 °C. The cells were passaged by trypsinization every 4 days and maintained in a 10 cm dish (BD Biosciences, Franklin Lakes, NJ, USA).

### Immunoprecipitation assays

#### Immunoprecipitation of endogenous GPNMB and NKA

Immunoprecipitation assays were performed using a Classic IP Kit (Thermo Fisher Scientific) according to the manufacturer’s instructions. NSC34 cells were plated at 1.4 × 10^5 ^cells/well in 6-well plates (BD Biosciences) and incubated overnight. The cell lysates were incubated with mouse anti-NKA α1 antibody, mouse anti-NKA α3 antibody, or normal mouse IgG (Santa Cruz Biotechnology). A mixture of equal parts of a protein sample and sample buffer with 20% 2-mercaptoethanol (Wako, Osaka, Japan) was subjected to SDS-PAGE (Wako). The separated proteins were then transferred onto a polyvinylidene difluoride membrane (PVDF, Immobilon-P; Merck KGaA, Darmstadt, Germany). To visualize the proteins on the membrane, the primary antibody was goat anti-GPNMB antibody (1: 500, R&D Systems Inc., Minneapolis, MN, USA) and the secondary antibody was horseradish peroxidase (HRP)-conjugated rabbit anti-goat antibody (1: 2000, Thermo Fisher Scientific). The immunoreactive bands were visualized using a chemiluminescent substrate (ImmunoStar^®^ LD; Wako). The band intensity was measured using an imaging analyzer (LAS-4000; Fuji Film, Tokyo, Japan).

#### Immunoprecipitation of the human recombinant GPNMB and NKA

NSC34 cells were treated with the human recombinant GPNMB (extracellular fragment; R&D Systems Inc.). The cell lysates were incubated with mouse anti-NKA α1 antibody, mouse anti-NKA α3 antibody, or normal human IgG (R&D Systems Inc.). To visualize the proteins on the membrane, the primary antibody was used normal human IgG (1: 500, R&D Systems Inc.). The secondary antibody was HRP-conjugated goat anti-human antibody (1: 50000, Santa Cruz Biotechnology).

### Immunostaining and confocal microscopy

#### Immunostaining of endogenous GPNMB and NKA

NSC-34 cells were seeded at 3.5 × 10^4 ^cells/well in a Lab-Tek II Chamber slide (Thermo Fisher Scientific) and incubated overnight. Then, cells were fixed in 4% paraformaldehyde (PFA) for 15 min. After blocking by 3% goat serum for 1 h, cells were incubated overnight at 4 °C with primary antibody, goat anti-GPNMB antibody (1:500), mouse anti-NKA α1 antibody, or mouse anti-NKA α3 antibody (1: 200). Subsequently, cells were incubated at room temperature with Alexa-488 or Alexa-568-labeled secondary antibody (1: 500, Thermo Fisher Scientific) for 1 h. Confocal images were obtained using Fluoview (Olympus, Tokyo, Japan).

#### Immunostaining of the human recombinant GPNMB and NKA

NSC-34 cells were transfected with 100 nM of mouse NKA α1 specific RNAi oligo or negative-control RNAi oligo (Nippon Gene Co., Ltd., Tokyo, Japan) by using Lipofectamine RNAiMAX (Thermo Fisher Scientific) for 48 h, according to manufacturer’s recommendations. Sequences included the NKA α1 small interfering RNA (siRNA) sense strand, 5′-CCAGUAACAUUCCGGAAAUdTdT-3′, and antisense strand, 5′-AUUUCCGGAAUGUUACUGGdTdT-3′. Then, cells were treated with the human recombinant GPNMB or PBS for 1 h, and fixed and blocked as the same as mentioned above. The cells were incubated overnight at 4 °C with primary antibody anti-human IgG antibody (1:500, R&D Systems Inc.), and mouse anti-NKA α1 antibody (1: 200). Subsequently, cells were incubated at room temperature with Alexa-488 or Alexa-546-labeled secondary antibody (1:500) for 1 h.

### Cellular membrane potential assay

NSC-34 cells were seeded at 7,000 cells/well into a 96 well black plate (BD Biosciences) and incubated overnight. Cells were then incubated for 1 h in Tyrode’s solution (132 mM NaCl, 5 mM KCl, 1 mM CaCl_2_, 1 mM MgCl_2_, 5 mM glucose, 5 mM HEPES, pH 7.4) containing 5 μM HLB021-152 (AnaSpec, Fremont, CA, USA) in a humidified atmosphere containing 5% CO_2_ at 37 °C. The fluorescent signal was measured by using a microplate reader by following the manufacturer’s instructions^23^.

### Cell death assay

NSC-34 cells were seeded at 7,000 cells/well into a 96-well plate (BD Biosciences) with DMEM without antibiotic drug and containing 10% FBS and incubated overnight. The cells were transfected with the empty vector (mock) plasmid or plasmid containing human mutant SOD1^G93A^ cDNAs containing the entire coding region, which were gifted from Dr. Gen Sobue, Nagoya University Graduate School of Medicine, by using Lipofectamine 2000 (Invitrogen) for 6 h. And then, the medium was replaced with DMEM containing antibiotic drug and 10% FBS and cells were cultured a further 42 h. Then, the medium containing NSC-34 cells transfected with human mutant SOD1^G93A^ cDNA was replaced with the serum-free DMEM and cells were treated with vehicle or the human recombinant GPNMB (2.5 μg/ml) for 24 h. NKA inhibitors, ouabain and sanguinarine, were used to investigated whether the neuroprotective effects of the GPNMB extracellular fragment were mediated by NKA. In the cell death assay using NKA α1 siRNA, 24 h after seeding into a 96-well plate, NSC-34 cells were transfected with the mock or human mutant SOD1^G93A^ cDNAs and 100 nM of mouse NKA α1 specific RNAi oligo or control RNAi oligo by using Lipofectamine 2000 with DMEM containing 1% FBS and without antibiotics, as described previously^14^. In general, in transfection with siRNA, DMEM without antibiotic drug and serum is used. However, when human mutant SOD1^G93A^ cDNAs were transfected together with siRNA, DMEM containing 1% FBS and no antibiotic drug were used. Then, NSC-34 cells were replaced with the serum-free DMEM and immediately treated with the human recombinant GPNMB (2.5 μg/ml) or vehicle for 24 h. Images were collected using an Olympus IX70 inverted epifluorescence microscope (Olympus). The total number of cells was counted and the percentage of PI-positive cells was used as a measure of dead cells, as described previously^46^. In a blinded manner, a total of at least 200 cells per condition were counted using image-processing software (Image-J ver. 1.33f; National Institutes of Health, Bethesda, USA).

### Western blotting

NSC34 cells were seeded at approximately 3.5 × 10^4^ cells per well in 24-well plates (BD Biosciences) and incubated for 24 h. The cells were treated with PBS or the human recombinant GPNMB. NKA inhibitors, ouabain and sanguinarine, were used to investigate whether the extracellular fragment of GPNMB activates the PI3K/Akt and MEK/ERK pathways through NKA. At the end of the culture period, NSC34 cells were washed with PBS once in order to remove dead cells. The NSC34 cells were then lysed using a RIPA buffer (50 mM Tris HCl, 150 mM NaCl, 0.5% sodium deoxycholate, 0.1% sodium dodecyl sulfate, and 1% Igepal CA-630) together with protease inhibitors and a phosphatase inhibitor cocktail (Sigma-Aldrich). The lysate was centrifuged and the supernatant was collected. Protein concentration was determined from a standard curve of bovine serum albumin (BSA) with a BCA protein assay kit (Thermo Fisher Scientific). A mixture of equal parts of a protein sample and sample buffer with 20% 2-mercaptoethanol was subjected to SDS-PAGE. The separated proteins were then transferred onto a PVDF. To visualize the proteins on the membrane, the following primary antibodies were used: rabbit anti-phospho-Akt (Ser473, 1: 1000), rabbit anti-Akt (1: 1000), rabbit anti-phospho-ERK 1/2 (1: 1000), rabbit anti-ERK 1/2 (1: 1000), rabbit anti-phospho-Src (Tyr416, 1: 500), and rabbit anti-Src (1: 500, Cell Signaling, Beverly, MA, USA). The secondary antibodies were HRP-conjugated goat anti-mouse and HRP-conjugated goat anti-rabbit antibody (1: 2000, Thermo Fisher Scientific). The immunoreactive bands were visualized using ImmunoStar^®^ LD. The band intensity was measured using LAS-4000.

### Statistical analysis

Data are presented as the means ± standard error of the mean (S.E.M.). Statistical comparisons were made using a two-tailed paired student’s *t*-test or one-way ANOVA followed by Dunnett’s test and Tukey’s test, with *p* < 0.05 indicating statistical significance.

## Additional Information

**How to cite this article**: Ono, Y. *et al*. Glycoprotein nonmetastatic melanoma protein B extracellular fragment shows neuroprotective effects and activates the PI3K/Akt and MEK/ERK pathways via the Na^+^/K^+^-ATPase. *Sci. Rep*. **6**, 23241; doi: 10.1038/srep23241 (2016).

## Figures and Tables

**Table 1:** Interacting proteins of GPNMB extracellular fragment.

Gel No.	Accession No.	MASDOT SCORE	Protein Name	Mass
MALDI-TOF-MS Assay				
No. 1	gil112363107	58.9	neurofilament medium polypeputide	95,998
No. 2	DASA_MOUSE	229	succinate dehydrogenase flavoprotein subunit, mitochondrial precursor	73,623
No. 3	VDAC_MOUSE	36	Voltage-dependent anion-selective channel protein 1	32,502
LC-FT-MS Assay				
No. 1	AT1A3_MOUSE	4196	Na^+^,K^+^-ATPase sabunit alpha-3	111,620
	AT2A1_MOUSE	2855	Sarcoplasmic/endoplasmic reticulum calcium ATPase1	109,355
	AT1A1_MOUSE	2478	Na^+^,K^+^-ATPase sabunit alpha-1	112,910
No. 2	DASA_MOUSE	5933	succinate dehydrogenase flavoprotein subunit, mitochondrial precursor	72,539
	CMC1_MOUSE	2458	Calcium-binding mitochondrial carrier protein Aralar1	74,523
	VATA_MOUSE	638	V-type proton ATPase catalytic subunit A	68,283
No. 3	VDAC1_MOUSE	2069	Voltage-dependent anion-selective	32,331
	VDAC2_MOUSE	922	Voltage-dependent anion-selective channel protein 1	31,713
	VDAC_MOUSE	799	Guanine nucleotide-binding protein subunit beta-2-like 1 channel protein 2	35,055
